# High Resistance of Potato to Early Blight Is Achieved by Expression of the *Pro-SmAMP1* Gene for Hevein-Like Antimicrobial Peptides from Common Chickweed (*Stellaria media*)

**DOI:** 10.3390/plants10071395

**Published:** 2021-07-07

**Authors:** Denis V. Beliaev, Natalia O. Yuorieva, Dmitry V. Tereshonok, Ilina I. Tashlieva, Marina K. Derevyagina, Alexei A. Meleshin, Eugene A. Rogozhin, Sergey A. Kozlov

**Affiliations:** 1 K. A. Timiryazev Institute of Plant Physiology RAS, 127276 Moscow, Russia; bdv@ippras.ru (D.V.B.); yuorieva@mail.ru (N.O.Y.); diman_ter_vi@mail.ru (D.V.T.); ii_tash@mail.ru (I.I.T.); 2 Russian Potato Research Center, 140052 Kraskovo, Russia; vzejruk@mail.ru (M.K.D.); a-mela@mail.ru (A.A.M.); 3 M.M. Shemyakin and Yu.A. Ovchinnikov Institute of Bioorganic Chemistry Russian Academy of Sciences, 117997 Moscow, Russia; serg@ibch.ru; 4 All-Russian Institute of Plant Protection, 196608 St.-Petersburg-Pushkin, Russia

**Keywords:** hevein-like antimicrobial peptides, *Solanum tuberosum*, genetic engineering, early blight, *Alternaria alternata*, *Alternaria solani*

## Abstract

In the common chickweed *Stellaria media*, two antimicrobial peptides (AMPs), SmAMP1.1a and SmAMP1.2a, have been shown to be proteolytically released as products of the expression of a single gene, *proSmAMP1*. In this study, the gene *proSmAMP1* was introduced into two potato varieties, Zhukovsky ranny and Udacha. These early-maturing varieties were shown to be susceptible to early blight caused by *Alternaria* spp. Most transgenic lines of either variety having strong expression of the target gene demonstrated high levels of resistance to *Alternaria* spp. during three years of cultivation, but did not otherwise differ from the initial varieties. Disease severity index (DSI) was introduced as a complex measure of plant susceptibility to early blight, taking into account the diameter of lesions caused by the *Alternaria* spp., the fungus sporulation intensity and its incubation period duration. Across all transgenic lines, the DSI inversely correlated both with the target gene expression and the copy number in the plant genome. Our results are promising for improving the resistance of potato and other crops to early blight by expression of AMPs from wild plants.

## 1. Introduction

Early blight is a worldwide-spread plant disease causing harm to potato, tomato and other cultivated *Solanum* species. As early as in the 19th century, the disease was shown to be caused by fungi of *Alternaria* spp., with *A. solani* and *A. alternata* being the most aggressive to *Solanum tuberosum*[1]. The fungi predominantly infect older tissues, and the disease symptoms start to appear at the lower leaves [2]. Hot and dry climate areas with alternating dry and rainy seasons are especially prone to early blight epiphytotics [3]. In such areas, potato yield losses averaged 29% and without crop protection, the losses reached 70–80% [4]. From 5 to 40% of the potato yield was lost because of early blight in Israel [5]. Timely application of fungicides is the most common system of crop protection against yield loss to early blight [6]. However, the prolonged application of fungicides to potato fields is known to lead to lower effectiveness of the fungicides [7] and to accumulation of toxic compounds in soil and potato tubers [8,9]. New early blight-resistant potato varieties are an effective alternative way to reduce yield losses today [2]. In an effort to obtain some sources of the early blight resistance genes for conventional breeding, wild *Solanum* species of various taxonomic groups were screened for disease resistance. Although *S. commersonii, S. neorossii* and *S. tarijense* Hawkes were the most resistant species, they turned out to be highly weatherdependent[10]. In another study, none of 280 interspecific hybrids resulting from the cross of *Solanum phureja* to *S. stenotonum* were found to be immune to *Alternaria* spp. These interspecific hybrids take decades to start producing large tubers, and they are difficult to cross with cultivated potato [11]. If wild potato species are used in breeding programs as sources of the early blight resistance genes, then their hybrid progeny often contain high amounts of teratogenic glycoalkaloids such as solanine and chaconine. Often, the resistant hybrids have low industrial value due to their late maturity and low yields [12]. Genetic engineering for resistance to early blight is an alternative to conventional breeding [13,14,15,16] and it is unlikely to modify the content of glycoalkakoids in potato.

The arsenal of plant defense against pathogens includes antimicrobial compounds of low molecular weight, so-called “small molecules” [17]. The latter, in turn, include antimicrobial peptides (AMPs) of several known classes encoded by DNA [18]. A number of studies reported transferring AMPs to potato. Hence, the plants of potato cv. Desire expressing the *MsrA3* gene for an antimicrobial cationic peptide became more resistant to *Fusarium solani*, a causative agent for dry rot, as well as to elevated temperatures (+33 °C) and wounding [19]. Transgenic potato plants harboring the synthetic gene *magaininD* for the peptide Magainin 11 from *Xenopus laevis* were resistant to soft rot caused by *Erwinia carotovora* subsp. *astroseptica* [20]. A variant of magainin was also shown to inhibit fungi. Hence, potato varieties Kufri Jyoti and Kufri Bahar expressed the gene *MSI-99* for a synthetic magainin and demonstrated resistance to *Aspergillus niger* in in vitro tests [21]. Potato’s own peptides of the snakin structural family demonstrated their antimicrobial properties. Snakin-1 inhibited growth of a ring rot causal agent of *Clavibacter michiganensis* subsp. *sepedonicus* as well as growth of the pathogenic fungi *Botrytis cinérea* and *Fusarium solani*, whereas Snakin-2 proved to be bactericidal to Gram-positive *Clavibacter michiganensis* subsp. *sepedonicus* and to Gram-negative *Sinorhizobium meliloti* bv. *Meliloti* species, and fungicidal to plant pathogens *B. cinerea*, *Fusarium solani* and *F. culmorum* [22]. The transformations of Belorussian potato varieties Odissey, Vetraz and Scarb with either *MsrA1* or *CEMA* genes for antimicrobial peptides of the cecropin-mellitin class improved plant resistance to *Phytophthora infestans*, *Alternaria solani* and *Erwinia carotovora* [23].

Common chickweed (*Stellaria media* L.) is a weed of the *Cariophyllaceae* family. It is a salad ingredient in UK and Ireland and a valuable medicinal plant since its extracts produce an anti-inflammatory effect [24]. The *S. media* peptide extracts inhibited growth and development of an oomycete *P. infestans* and a fungal pathogen *Fusarium oxysporum* in vitro [25]. We have already characterized SmAMP3, a hevein-like peptide from *S. media* leaves that has specific antifungal activity against many species of fungal plant pathogens that contain chitin in their cell wall architectures [26]. Additionally, using high-throughput next-generation sequencing of *S. media* transcriptome, we collected and analyzed a library of ESTs potentially encoding some members from the six known plant AMP families—alpha-hairpinins, defensins, hevein-like peptides, lipid-transfer proteins, snakins and thionins [26,27]—that opened novel possibilities to study the molecular diversity of AMPs from the common chickweed at the proteomic level.

In the search for the *S.media* mechanisms of fungal resistance, the *proSmAMP1* gene was isolated from its seeds. It encodes two hevein-like peptides, SmAMP1.1a and SmAMP1.2a, that are proteolytically released from the proSmAMP1nascent peptide (NCBI GenBank: FN663151.1). Expression of this gene may have a potential advantage to increase the number of AMP species being introduced in comparison to single peptide-coding genes. Additionally, the transgenic tobacco and *Arabidopsis* plants expressing the *proSmAMP1* gene already demonstrated resistance to plant pathogens *Bipolaris sorokiniana* and *Thielaviopsis basicola*[28].

In the study reported herein, the activities of the SmAMP1.1a and SmAMP1.2a peptides against fungi of *Alternaria* spp. are shown, the *proSmAMP1* gene is expressed in potato varieties having different levels of resistance to *Alternaria* spp. and the response of the transgenic plants to this pathogen is analyzed.

## 2. Results

### 2.1. The Activities of the S. media Peptides Against Alternaria alternata

The hevein-like antimicrobial peptides SmAMP1.1a and SmAMP1.2a were purified from *S.media* and tested for their activities against *Alternaria alternata*. The minimal inhibitory concentrations of SmAMP1.1a and SmAMP1.2a were 3.5 and 3.0 µM, respectively. Fifty percent inhibition of fungal growth by these peptides was achieved with 8.6 µM of SmAMP1.1a or 7.1 µM of SmAMP1.2a. When compared to the published data (see Discussion), the relatively high activities of the SmAMP1.1a and SmAMP1.2a peptides led us to assume that their expression in potato would alter the reaction of this crop to *Alternaria alternata*.

### 2.2. Selection of the Varieties According to Their Disease Severity Indices (DSI) for Genetic Transformation by the S. media ProSmAMP1 Gene 

The early blight susceptibility of potato varieties available to us wasmeasured in order to select the most susceptible varieties that were likely to respond to AMP expression and to have the reference values for comparison with those of the transgenic plants. A complex criterion, named the disease severity index *(DSI)*, was coined to characterize the plant genotype susceptibility to early blight [29]. It takes into account the average lesion diameter in millimeters, the sporulation intensity score and the incubation period duration; that is, the number of days from inoculation until the appearance of the first disease symptoms (see the Materials and Methods). 

**Figure 1:**
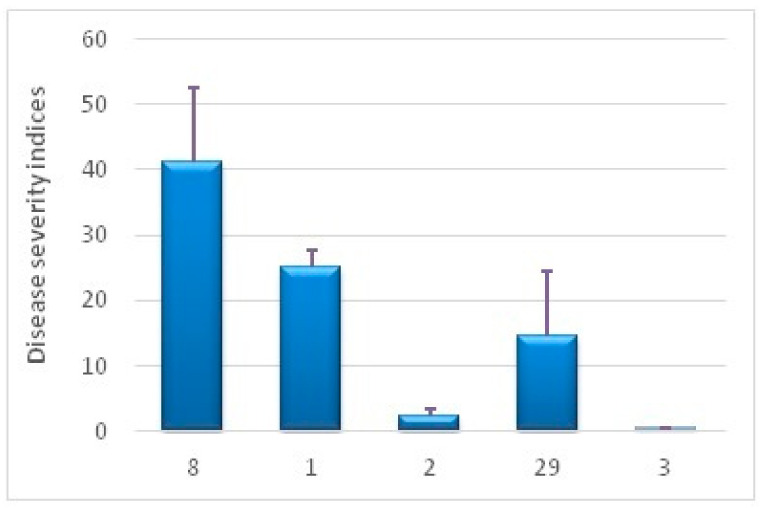
Susceptibility of potato varieties to early blight measured in disease indices. The varieties used were: 8, Udacha; 1, Zhukovsky ranny; 2, Yubiley Zhukova; 29, Skoroplodny; 3, Nikulinsky.

The varieties used for this test were Zhukovsky ranny, an ultra-early variety that matures in less than 60 days; Udacha, Skoroplodny and Yubiley Zhukova, early varieties maturing in 60 to 70 days; and Nikulinsky, amedium-maturing variety maturing in 100–110 days [30]. Our test results refer to Udacha and Zhukovsky ranny as highly susceptible varieties, with their DSI being much higher than 15. According to our results, Skoroplodny is a medium-susceptible variety, and Yubiley Zhukova and Nikulinsky are the resistant ones (Figure 1). More varieties are to be tested to find if the DSI and early maturity correlate. The raw data of the infection assay are in ESM Table S1. Based on their maturity types and early blight susceptibility data, Zhukovsky ranny and Udacha were chosen for genetic transformation to find out if their resistance to early blight could be improved by *proSmAmp1* expression.

### 2.3. Potato Transformation with ProSmAMP1

**Figure 2:**
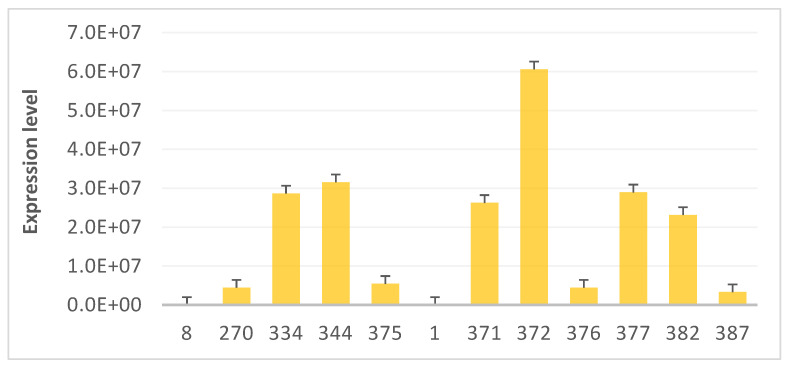
PCR analysis data of *ProSmAMP1* transgene expression in transformants 270, 334, 344 and 375 derived from variety Udacha (8) and in transformants 371, 372, 376, 377, 382 and 387 derived from variety Zhukovsky ranny (1). Lines 371 and 372 of Zhukovskii ranny variety and lines 270 and 344 of Udacha variety are shown in orange since the product of *ProSmAMP1* in these lines was confirmed by proteomics.

The *pBI-proSmAMP1* construct for potato transformation was a kind gift from A.V. Babakov of the All-Russia Institute of Agricultural Biotechnology, Moscow. It carries the *proSmAMP1* gene under *CaMV* 35S promoter [28]. The leaf explants of 4-week-old aseptic plants of Zhukovsky ranny and Udacha varieties were used for transformation according to [31]. The regenerated plants that withstood 50 mg/L Km in selection medium and had green shoots and well-developed roots were analyzed byPCR for the absence of *Agrobacterium* with the primers specific for *virE2* gene. The latent agrobacterial contamination was detected in fewer than 8% of the regenerated plants propagated on antibiotic-free media. The target gene was then detected by PCR in 34 transformed lines of Zhukovsky ranny variety, and the target gene expression was demonstrated by RNA hybridization in 12 lines. The transformation of the Udacha variety produced six PCR-positive lines, with all of them expressing the target gene. Based on the PCR analysis, the transformation efficiencies per initial explant were 54% for Zhukovsky ranny and 12% for Udacha. The lines 270, 334, 344 and 375 of Udacha background (8) and the lines 371, 372, 376, 377, 382 and 387 of Zhukovsky ranny background with different levels of the transgene expression (Figure 2) were chosen for further analysis. 

**Figure 3:**
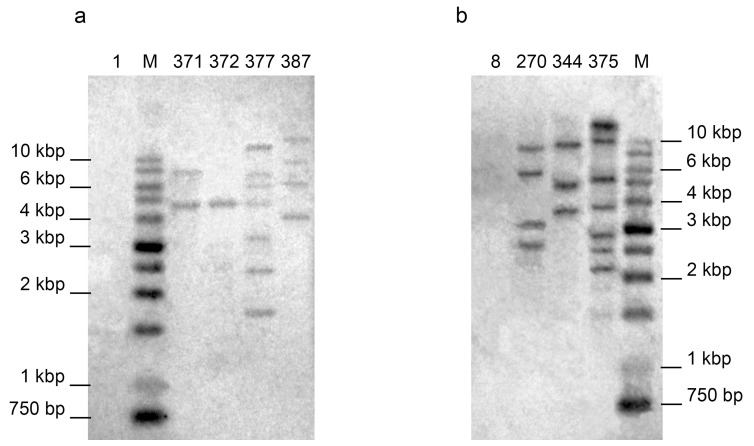
Southern hybridizations of HindIII-digested genomic DNA of potato transformants of (**a**) Udacha and (**b**) Zhukovsky ranny varieties. Of Udacha, 8, 270,342,344 and 375, initial variety and the transformed lines; of Zhukovsky ranny, 1, 371,372,377 and 378, initial variety and the transformed lines.

Integration of the *proSmAMP1* gene into genomes of these lines was confirmed by Southern hybridization (Figure 3 and Electronic Supplementary Material (ESM) Figure S1), and the lines were grown in pots in a greenhouse during the summers of 2012, 2014 and 2015. No differences in morphology and tuber production were observed between transgenic and initial lines of either variety.

### 2.4. Resistance of the Transgenic Plants to Early Blight

**Figure 4:**
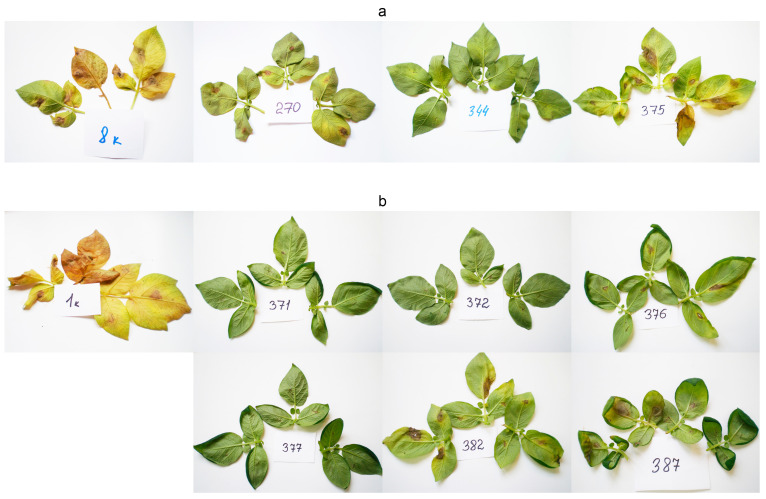
Early blight symptoms in the *Alternaria* spp. inoculated leaves of initial potato varieties and their respective transgenes: (**a**) Udacha and its transgenic lines 270, 344 and 375. Photos were taken in 2015; (**b**) Zhukovsky ranny and its transgenic lines 371, 372, 382, 376, 377 and 387. Photos were taken in 2016.

**Figure 5:**
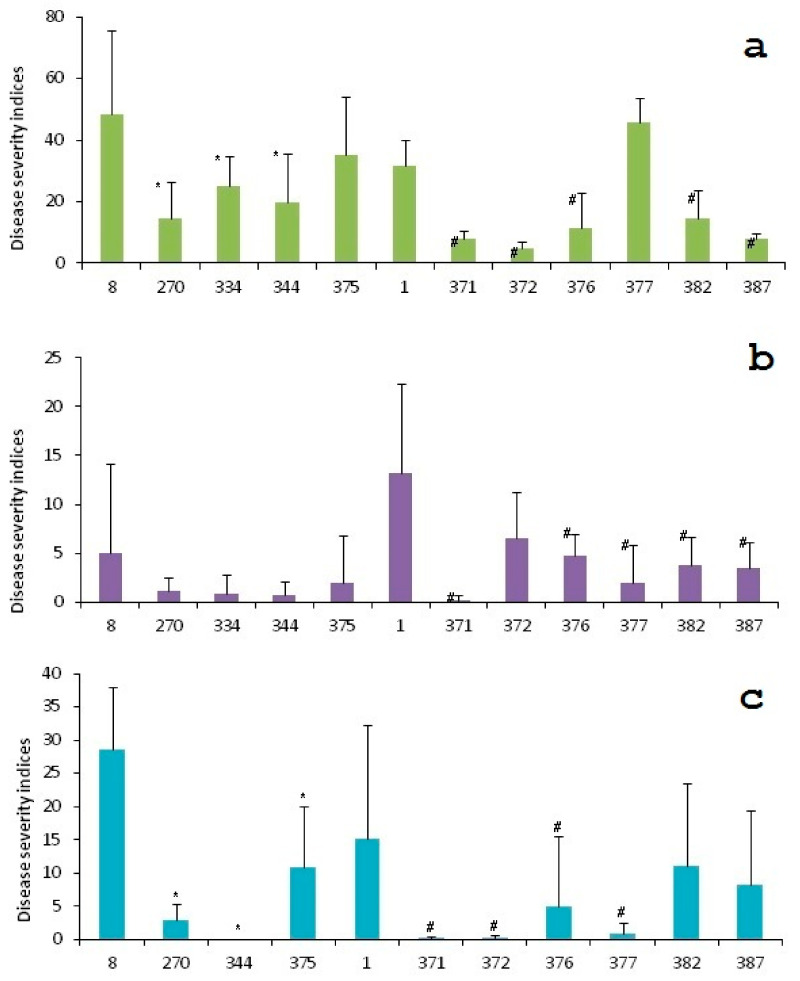
Disease severity indices (*DSI*) of the transformants inoculated with Alternaria spp. The inoculations were performed in 2012 (**a**), 2014 (**b**) and in 2015 with the Udacha transformants and in 2016 with the Zhukovsky ranny transformants (**c**). * and # designate lines significantly different from the initial variety (*p* < 0.05).

Detached leaves of all transgenic lines and the initial varieties were inoculated with *Alternaria solani* and *Alternaria alternata* conidia and assayed for resistance to early blight during the summers of 2012, 2014, 2015 and 2016. See Table S1 for the raw data and Figure 4 for images of the infected leaves, taken in 2015/2016. The initial Zhukovsky ranny and Udacha varieties were severely damaged, while their respective transgenic lines showed markedly less damage. The disease severity indices *(DSI)* of all lines are given in Figure 5. Both initial varieties remained highly susceptible regardless of the year, and all lines almost always had a smaller *DSI* than the initial variety. All lines demonstrated consistency in their *DSI* relative to the initial line and to each other for all three years of measurements. Concerning the Udacha variety (designated as 8 in Figure 4), its line 375 had moderate if any improvement in resistance compared to the initial variety, whereas the other transformed Udacha lines were always better than line 375 and classified as resistant (*DSI* smaller than 1) for two years: 2014 and 2015. 

The Zhukovsky ranny transformants performed even better; the lines 376, 382 and 387 were medium resistant, and the lines 371, 372 and 377 are to be tested further to prove that they are highly resistant. The further tests are needed, firstly because the high level of resistance to early blight is an achievement, and secondly, the DSI of line 377 stood out in 2012. Additionally, as judged by *DSI,* both overall susceptibility of potato and of the individual transgenic and untransformed lines varied from year to year, apparently because of unstable plant growth conditions. In this study, potato plants were grown in a greenhouse with only their water status being controlled (Figure 5). During plant growth, the air temperature and humidity in the greenhouse varied greatly from year to year. The growth conditions of individual plants, sometimes of the same line, were also variable, resulting in large experimental error bars in Figure 5. That instability of growth condition apparently resulted in a *DSI* calculated in 2012 for the line 377 being worse than that of the initial untransformed line.

A negative correlation between the *proSmAMP1* mRNA levels and the disease severity indices of the respective transgenic lines were observed in all three years: overall, the higher the target gene expression in a given plant line, the lower that line’s plant disease severity index. Averaged over three years, the correlation coefficients were −0.50 ± 0.20 for the Zhukovsky ranny transformants and −0.43 ± 0.29 for the Udacha transformants. The line 377 data from 2012 were omitted from the analysis (see above). The year-specific correlation coefficients are given in Table S2. 

Southern hybridization of the transgenic plant DNA with the *proSmAMP1* probe detected from 1 to 7 copies of the transgene integrated in a genome, whereas no signals were detected in the gel lanes with genomic DNA from untransformed plants (see Figure 3). Additional proof of specificity was the Southern hybridization of potato DNA cut with *HindIII* and *EcoRI*, the enzymes with recognition sites within the T-DNA, so the hybridizing fragment was of precise size, of 1913bp (ESM Figure S1). Averaged over three years of measurements, the negative correlation coefficients between the transgene copy number and the plant disease severity index were −0.65 ± 0.16 for the Zhukovsky ranny transformants and −0.52 ± 0.14 for the Udacha transformants. The line 377 data from 2012 were omitted from the analysis (see above) and the lines 334, 376, 382 and 387 were not analyzed by Southern hybridization. The year-specific correlation coefficients are given in Table S2. 

## 3. Discussion

The action of two *S. media* hevein-like antimicrobial peptides on *Alternaria* spp. fungi in vitro and in vivo is reported here. As little as 3 µM of these peptides inhibited fungal growth in vitro. The antifungal action of this class of AMPs is associated with binding to chitin, the main compound of filamentous fungal cell wall [27]. However, 10-cysteine hevein-like peptides belonging to the WAMP subfamily [32,33] also have an alternative mode of antifungal action. The peptides inhibited Znmetalloproteases secreted by *Fusarium* spp. as factors of virulence [34,35,36]. Out of many AMPs of other classes, only a handful demonstrated high activity against *Alternaria* spp. For instance, thanatin was shown to be active against bacteria in the 1.5–12 µM range [37] and against fungi in the submicromolar range [38]. Cecropins are another family of peptides with reported high activities against *Alternaria* spp. (see [39] for an example). Both thanatin and cecropins are from insects, and so they may not have priority in practical applications over plant AMPs. As for the latter, a cysteine-rich peptide from pine [40] was shown to be active in the submicromolar range, a peptide from foxtail millet was active at the micromolar range [41] and two AMPs named aracins were reported to inhibit *Alternaria* spp. in in vitro assays in concentrations below 1 µM [42]. Concerning the activity of plant AMPs derived from some other families, strong inhibition by defensins of D-type was reported, for instance, by RsAFP1/2 from *Raphanus sativus* seeds and by Ns-D1/2 from *Nigella sativa* seeds [43,44].

Following evaluation of the *S. media* peptides for their activities against *Alternaria* spp., their genes were introduced in potato. To choose potato varieties for the genetic transformation, several criteria were to be met. The varieties were to be amenable to agrobacterial transformation, preferably agriculturally important, that is, either widely grown or easy to be crossed with other varieties, and susceptible to early blight. The varieties used here had high (54 and 12%) transformation efficiencies and were ultra early and early. Early and medium-mature varieties are widely grown in Russia due to its climate, and our results herein confirm the field tests described elsewhere about such varieties being the most sensitive to early blight [45,46,47]. Several varieties available to us that fit the other criteria were evaluated for their early blight resistance.

Our measure of early blight symptoms, named DSI, is described in the Materials and Methods, for we believe the method of obtaining it [29] has not been published in English. DSI, the Disease Severity Index, combines three early blight symptoms observed in the detached leaves that were artificially inoculated with the *Alternaria* spp. conidia suspension. This index is extensively used in Russian Potato Research Center to characterize potato interaction with *Alternaria* spp. Elsewhere, the relative square leaf area of *Alternaria* spp. infection, given as % of the damaged leaf area [48], or the disease severity ranging from 1 to 7 or from 1 to 4 corresponding to lesions from 0 to more than 5 mm, respectively, have been used to judge the early blight severity [4,49,50,51,52]. Different complex criteria taking into account various parameters of disease progression such as incubation period length, spore production and lesion expansion rates have been used elsewhere as well [30,53,54].

Using DSI, several varieties were screened and the most susceptible varieties, Zhukovsky ranny and Udacha, were used for transformation with an antifungal gene proSmAMP1 driven by 35S *CaMV* promoter. The expression of the transgene in the obtained plant lines was quantified by RNA hybridization, and the transgenic plants, in turn, were assayed for resistance to early blight using the DSI technique. Some images of inoculated leaves are given in Figure 4. There, the initial Udacha variety and line 375 with low transgene expression have large chlorotic and necrotic areas, whereas the disease symptoms are almost absent in line 344 of the Udacha variety with high target gene expression. Zhukovsky ranny transgenic lines 371, 372 and 377 with high expression of SmAMP1 also had almost undetectable necroses and chloroses. The lines 376, 378 and 382 with lower transgene expression had rather considerable symptoms of early blight, and on the untransformed Zhukovsky ranny plants the disease symptoms were clearly seen. The data on the response of the transgenic plants to early blight collected for three years (Figure 5) consistently point to the reciprocal relationship of SmAMP1 expression and DSI with the strongest ProSmAMP1-expressing transgenic plants, demonstrating the outstanding resistance to early blight. The corresponding correlation coefficients were always negative (see Results). The reasons for these transgene expression/DSI coefficients not being very close to 1 may be the instability of the growth conditions, a complex relationship with the *ProSmAMP* transcription, RNA turnover, translation, post-translational modification, intra- and extracellular transport, peptide–pathogen interactions and the somaclonal variation that often accompanies genetic transformation [55]. Along with genotype, growth conditions seriously affect early blight development in potato. Temperature, rather than relative air humidity or precipitation, has the greatest impact here [56,57]. An earlier study with ProSmAMP1 gene transfer to plants demonstrated the effect of its expression on the resistance to the fungal pathogens *Bipolaris sorokiniana* and *Thielaviopsis basicola* conferred upon A. thaliana and tobacco [28]. In a related study of potato varieties transformed with the *proSmAMP2* gene, no correlation of the target gene expression with the pathogen resistance of the transgenic plants was reported [58]. Similar trends were reported elsewhere, but not all authors expressed it numerically. For example, expression of *rolB* gene inversely correlated with resistance of the transgenic plants to *A. solani* and *F. oxysporum* [59]. Transgenic olives *Olea europaea* expressing the antifungal protein gene *Afp* from *Aspergillus giganteus* had the transgene expression inversely correlated with the symptoms of white root rot caused by *Rosellinia nacatrix* in roots and leaves, with the correlation coefficients being −0.968 and −0.954, respectively [60]. However, when transgenic *Vigna mungo* expressing the *ChiB* gene of a bacterial chitinase was infected by *Erysiphae polygoni*, a causative agent for powdery mildew, the correlation of the chitinase activity with the rate of disease growth was low; the correlation coefficient was −0.1726 [61].

In this study, the calculated correlation of the transgene copy number and the disease severity index was also negative. One explanation may be the additive effect from several transgenes integrated in different regions of the potato genome with overlapping expression patterns, so that the transgene is expressed under a wider spectrum of conditions. Other authors also attempted to relate the target gene copy number and the values of specific traits, including plant pathogen resistance. For instance, commercial potato varieties carrying the *RB* gene from *Solanum bulbocastanum* were more resistant to late blight during two years of trials, and the resistance was higher in plants having higher transgene copy numbers, up to 15 copies. The latter plants also had high *RB* transcript content [62].

## 4. Materials and Methods

### 4.1. Isolation and Identification of the SmAMP Peptides from S. media 

Screening and isolation of the SmAMPs from *S. media* was conducted according to the method described earlier [26].

### 4.2. Antifungal Assays

Antifungal activities of the peptides were determined as described in [32]. Inhibitions of spore germination were estimated spectrophotometrically by absorption at 620 nm in a microtiter plates reader Spectramax250 (Molecular Devices, San Jose, CA, USA) after incubations of spore suspension (2 × 10^4^ conidia/mL) with the peptide solutions for 48 h at 22 °C. IC_50_ values showing the peptide concentrations required for 50% inhibition of growth were calculated as the results of 5 independent measurements. Inhibition of hyphae elongation and morphological changes in the fungi were examined by light microscopy.

### 4.3. Assessment of Plant Resistance to Alternaria spp. Disease Severity Index (DSI)

For the detached leaf assays, plants were grown in pots in a glasshouse in a substrate composed of 4 parts of Vozdyushnyi mix (Eco-AgTi, Schelkovo, Russia) and 1 part of perlite until flower buds formed (for 40–55 days). The plants were grown in a glasshouse under natural sunlight and watered daily with neither air temperature nor humidity being controlled. For the fungus resistance assays, three terminal leaflets from the fully developed leaves at the middle part of the stem were detached from each of three plants of selected potato lines, thus giving nine samples for each data point. The leaflets were then placed on wet filter paper in trays adaxial side down, and the leaf surfaces were wounded by a glass rod and inoculated by 100 µL of a 10-day old culture of 2–3 × 10^4^ conidia/mL of *A. solani* str. A14SdL3 and 3–5 × 10^4^ conidia/mL of *A. alternata* str. F-34. The strains were obtained from the “Myxomycetes Spores Collection” of the Mycology and Algology Department, Biology Faculty, Lomonosov Moscow State University, Moscow, Russia. The inoculums were prepared immediately before the inoculation according to [63]. The trays with inoculated leaves were covered by glass for higher humidity, and the inoculum drops were removed after 24 h. The leaves were incubated at 18–20 °C and vented daily for 8 days. The disease symptoms were registered daily. For each inoculation, the incubation period length *IPL_i_*was recorded as the number of days from inoculation until the appearance of the first disease symptoms. After 8 days, the lesion diameters *LD_i_* in millimeters and the sporulation intensity *SI_i_* scores were also recorded, with the scores 1, 2 and 3 assigned to weak, medium and abundant sporulations, respectively. A complex disease severity index, *DSI*, was coined to characterize the reaction of potato lines to the *Alternaria* spp. attack. The *DSI* takes into account parameters related to the disease severity *LD_i_*and SI_i_, and the incubation period length *IPL_i_* related to the time a genotype can withstand the pathogen attack:

$$DSI=\frac{1}{IN}\sum_{i=1}^{IN}\frac{LD_{i}\times SI_{i}}{IPL_{i}}$$

where *DSI* is the disease severity index; *LD_i_* is a lesion diameter (mm); *SI_i_* is the sporulation intensity score; *IPL_i_* is the incubation period length in days; and *IN* is the number of inoculations [29]. If no symptoms of disease infection were observed on the 8th day post inoculation, the DSI was assigned to be 0.

Based on the *DSI*, the genotypes were classified as follows: genotypes with *DSI* = 0–1.0 were highly resistant, with *DSI* = 1.1–5.0 they were resistant, with *DSI* = 5.1–10.0 they were medium resistant, with *DSI* = 10.1–15.0 they were susceptible and with *DSI* over 15 they were highly susceptible.

### 4.4. Plant Genetic Transformation

The aseptic potato plants of Zhukovsky ranny and Udacha varieties from the “Bank of virus-free potato cultivars” of Russian Potato Research Center, Kraskovo, Russia were grown in glass tubes on basal MS medium [64] with 2% sucrose and 0.7% agar-agar at 22–24 °C under 150 µmol m^−2^ s^−1^ of white light on a 16/8 cycle. After 4 weeks, the leaf explants were used for *Agrobacterium*-mediated transformation with *pBI-proSmAMP1* [28] according to [31]. The regenerated plants were subcultured every 4 weeks on selection medium composed of MS with 2% sucrose, 0.7% agar-agar and 50 mg/L kanamycin. The green rooted plants free of bacteria were used for further analyses.

### 4.5. Molecular Analyses of the Transformed Plants

Potato genomic DNA was isolated with CTAB (Stewart and Via 1993). The primers AMPSh-F 5′-CTTACATACAAAAGCTAGTCAC-3′ and AMPSh-R 5′-TCATTCCATAGACTTGTTTATGA-3′ were used for amplification of the 765bp long fragment of the *proSmAMP1* gene from genomes of the transformed potato plants. To confirm the absence of *Agrobacterium*, the transgenic plant DNA was amplified with forward 5′-CGAATACATTCTCGTGCGTCAAAC-3′ and reverse 5′TTTCGAGTCATGCATAATGCCTGAC-3′ primers for the *virE2* gene of *Agrobacterium*. Following the initial denaturation at 94 °C for 2 min, 35 cycles of the PCR program consisted of denaturation at 94 °C for 30 s, annealing at 60 °C for 30 s and synthesis at 70 °C for 30 s, performed in Tertsik (ZAO DNK-Tekhnologia, Russia). The PCR products were separated in 1.2% agarose/TAE gel with 1 Kb DNA Ladder (Sibenzyme, Russia) and visualized by staining in 0.5 mg/L EtBr. Total RNA isolation from leaves and Northern hybridization was performed according to [65], except the probe was the *BamHI-SacI* fragment of *pBI-proSmAMP1* containing the *proSmAMP1* gene. For Southern hybridizations, genomic DNA was isolated according to [66], then 30 µg of DNA from each plant was digested with either *HindIII* alone or *HindIII* and *EcoRI*, separated in a 1% agarose/0.5X TAE gel, transferred to the HybondN+ membrane (GE Healthcare, Chicago, IL, USA) and hybridized to the *BamHI-SacI* fragment of *pBI-proSmAMP1* containing the *proSmAMP1* gene according to the protocol provided by the membrane’s manufacturer. The Northern and Southern hybridization signals were detected using PhosphoImager (GE Healthcare, Chicago, IL, USA) hardware and software.

### 4.6. Statistical Analysis

Mean values with their standard deviations, correlation coefficients and Student’s *t*-test *p*-values (r) for two variables were computed with Microsoft Excel.

## 5. Conclusions

Finally, it should be noted that there are a variety of transgenic crops available in markets in many countries, and, partially, they possess high resistance to biotic stress factors, predominantly to insect pests, based on entomopathogenic bacterial endotoxins from *Bacillus thuringiensis*. There has not been achieved any real success to create gene-modified (GM) plants with confirmed resistance to bacterial or fungal diseases at the molecular level. We believe that our studies can promote the application ofplant antimicrobial peptides from wild plants to make them effective inside cultivated plants. This is the basis of so-called “cis-genic” plants, when a single or multiple gene(s)is (are) transferred from plant to plant. The results obtained in this study show that expression of the *proSmAMP1* gene from *S. media* improved the early blight resistance of early-maturing commercial potato varieties. Two *ProSmAMP1*transgenic lines per each of the two varieties transformed consistently demonstrated high resistance to *Alternaria* spp. infection. Relative to the initial varieties, the resistance of the best transgenic lines was about an order of magnitude better, and these lines are promising for further use in potato breeding. At last, GM crops expressing plant AMPs are going to be considered for commercial production in the future, because they have resistance to economically important fungal diseases, in such a way to suppress yield losses. To the advantage of biosafety, AMPs are represented by polypeptides, which are proteolytically degraded inside the human digestive tract in the same manner as any other protein.

## Data Availability

Data are contained within the manuscript.

## Supplementary Materials

The following are available online at https://www.mdpi.com/article/10.3390/plants10071395/s1, Figure S1: Southern hybridizations of *HindIII* and *EcoRI*-digested genomic DNA of potato transformants of (**a**) Udacha and (**b**) Zhukovsky ranny varieties., Table S1: Raw data of potato infection assays for the years 2011, 2012, 2014, 2015 and 2016, Table S2: Correlation coefficients between either ProSmAMP1 expression or copy number and DSI, per year.

## Special Note

This paper is dedicated to the late Prof. E. V. Grishin and Prof. Ts. A. Egorov. Their contribution to this research will take us all a long time to truly appreciate.
