# Supplementary material for: High Resistance of Potato to Early Blight Is Achieved by Expression of the Pro-SmAMP1 Gene for Hevein-Like Antimicrobial Peptides from Common Chickweed (Stellaria media)
Source: Plants (Basel). 2021 Jul 7;10(7):1395. doi: 10.3390/plants10071395 (PMC8309211; doi:10.3390/plants10071395)
Supplement: Supplementary file 1 [file plants-10-01395-s001.zip › Table S1.pdf]

Table S1. Raw data of potato infection assays for the years 2011, 2012, 2014, 2015 and 2016.

2011

Number of initial potato varieties	Incubation period length (IPLi)	Sporulation intensity (SIi)	Lesion diameter (LDi)	Disease severity index (DSIi)
8	3	3	40	40
	2	3	40	60
	2	3	22	33
	3	3	35	35
	2	3	35	52,5
	2	3	30	45
	3	3	31	31
	3	3	25	25
	2	3	33	49,5
1	2	2	22	22
	2	2	27	27
	2	2	24	24
	2	2	23	23
	2	2	25	25
	2	2	25	25
	2	2	22	22
	2	2	28	28
	2	2	29	29
2	4	1	10	2,5
	5	1	10	2
	3	1	13	4,333
	5	1	10	2
	3	1	10	3,333
	5	1	11	2,2
	7	1	2	0,286
	7	2	6	1,714
29	3	3	25	25
	5	1	25	5
	6	1	25	4,167
	6	2	20	6,667
	3	2	20	13,333
	6	2	20	6,667
	2	3	21	31,5
	2	2	23	23
	3	2	23	15,333

2011 continued from page 1

3		0	0	0
	7	1	3	0,429
		0	0	0
		0	0	0
		0	3	0
	7	1	0	0
		0	0	0
	7	1	5	0,714
		0	0	0

2012

Number of initial potato varieties	Incubation period length (IPLi)	Sporulation intensity (SIi)	Lesion diameter (LDi)	Disease severity index (DSIi)
8	2	3	50	75
	3	3	50	50
	3	3	50	50
	2	3	50	75
	3	3	52	52
	3	3	70	70
	3	1	2	0,667
	4	3	20	15
270	3	1	40	13,333
	3	1	35	11,667
	3	1	30	10
	3	1	40	13,333
	3	2	40	26,667
	3	2	60	40
	3	1	20	6,667
	4	1	30	7,5
	7	1	3	0,429
334	3	2	40	26,667
	3	3	40	40
	7	2	40	11,429
	3	3	30	30
	3	2	20	13,333
	4	2	30	15
	3	3	30	30
	3	3	30	30
	3	3	30	30

2012 continued from page 2

344	3	1	20	6,667
	3	1	30	10
	3	2	60	40
	3	3	50	50
	3	2	30	20
	4	2	25	12,5
	3	2	30	20
	3	2	30	20
	7	1	1	0,143
375	3	2	60	40
	3	3	55	55
	3	2	40	26,667
	3	3	45	45
	7	1	3	0,429
	2	3	35	52,5
	4	3	35	26,25
1	3	2	50	33,333
	3	3	40	40
	3	2	45	30
	3	3	40	40
	3	3	35	35
	3	3	40	40
	3	3	30	30
	3	3	20	20
	4	2	35	17,5
371	3	1	30	10
	3	1	15	5
	4	1	40	10
	3	1	15	5
	3	1	35	11,667
	3	1	23	7,667
	3	1	30	10
	3	1	15	5
	4	1	20	5
372	4	1	20	5
	4	1	10	2,5
	5	1	10	2
	3	1	20	6,667
	4	2	15	7,5
	5	1	20	4
	3	1	25	8,333
	5	1	25	5
	5	1	15	3

2012 continued from page 3

376	3	1	45	15
	3	1	40	13,333
	3	3	40	40
	3	1	20	6,667
	3	1	20	6,667
	4	1	30	7,5
	4	1	20	5
	4	1	20	5
	4	1	20	5
377	3	3	55	55
	3	3	43	43
	3	3	40	40
	3	3	55	55
	3	3	50	50
	3	3	50	50
	3	3	50	50
	3	3	35	35
	3	3	35	35
382	3	1	40	13,333
	3	2	45	30
	3	1	40	13,333
	3	1	30	10
	3	2	40	26,667
	7	1	40	5,714
	3	1	40	13,333
	4	1	1	0,25
	5	2	40	16
387	3	1	20	6,667
	3	1	30	10
	7	1	30	4,286
	4	1	30	7,5
	4	1	30	7,5
	4	1	30	7,5
	3	1	30	10
	3	1	30	10
	5	1	30	6

2014

Number of initial potato varieties	Incubation period length (IPLi)	Sporulation intensity (SIi)	Lesion diameter (LDi)	Disease severity index (DSIi)
8	5	0	35	0
		0	0	0
	5	2	12	4,8
	5	0	30	0
		0	0	0
		0	0	0
	4	1	35	8,75
	4	3	37	27,75
	5	2	10	4
270	5	0	10	0
		0	0	0
	5	2	6	2,4
	7	1	6	0,857
	7	2	10	2,857
		0	0	0
	6	2	10	3,333
	7	1	5	0,714
	8	0	2	0
334	4	0	7	0
	8	0	2	0
	8	0	2	0
		0	0	0
	7	2	7	2
	7	2	20	5,714
		0	0	0
		0	0	0
		0	0	0
344	8	0	2	0
	8	0	2	0
		0	0	0
	6	1	13	2,167
		0	0	0
		0	0	0
	5	2	10	4
		0	0	0
		0	0	0

2014 continued from page 5

375		0	0	0
	5	1	10	2
	2	1	30	15
		0	0	0
		0	0	0
		0	0	0
		0	0	0
		0	0	0
		0	0	0
1	5	3	12	7,2
	6	3	12	6
	7	3	15	6,429
	5	3	35	21
	4	3	40	30
	5	3	40	24
	5	3	10	6
	4	3	13	9,75
	5	3	14	8,4
371		0	0	0
		0	0	0
		0	0	0
		0	0	0
		0	0	0
	7	0	2	0
		0	0	0
	7	1	10	1,429
		0	0	0
372	6	3	20	10
	7	1	12	1,714
	7	1	10	1,429
	4	1	30	7,5
	6	1	10	1,667
	6	2	12	4
	4	1	30	7,5
	5	2	35	14
	4	3	15	11,25
376	7	1	30	4,286
	8	1	20	2,5
	7	1	20	2,857
	6	1	30	5
	7	1	35	5
	6	1	20	3,333
	6	1	30	5
	6	2	30	10
	6	1	30	5

2014 continued from page 6

377	8	0	2	0
	5	3	20	12
	6	2	7	2,333
		0	0	0
	5	1	13	2,6
	8	0	5	0
		0	0	0
		0	0	0
		0	0	0
382	3	2	13	8,667
	3	2	10	6,667
	5	1	10	2
	4	0	3	0
	3	2	4	2,667
	3	1	3	1
	5	1	13	2,6
	5	1	13	2,6
	6	3	14	7
387	8	1	30	3,75
	5	1	30	6
	8	1	30	3,75
	5	1	30	6
	6	1	30	5
	5	1	30	6
	8	0	2	0
	7	1	6	0,857
	8	0	3	0

2015

Number of initial potato varieties	Incubation period length (IPLi)	Sporulation intensity (SIi)	Lesion diameter (LDi)	Disease severity index (DSIi)
8	2	2	30	30
	2	2	40	40
	2	2	30	30
	2	3	10	15
	2	3	10	15
	2	3	15	22,5
	2	3	20	30
	2	3	25	37,5
	2	3	25	37,5

2015 continued from page 7

270	3	2	10	6,667
	4	2	10	5
	4	2	10	5
	3	1	5	1,667
	3	2	8	5,333
	3	2	10	6,667
	2	1	8	4
		0	0	0
	4	1	10	2,5
	3	1	10	3,333
	3	1	10	3,333
	7	0	1	0
	3	1	10	3,333
	3	1	10	3,333
	4	0	10	0
	5	0	1	0
	5	0	1	0
344	4	0	1	0
	4	0	2	0
	4	0	1	0
	6	0	1	0
	5	0	1	0
	5	0	1	0
	7	0	1	0
	6	0	5	0
	7	0	1	0
375	2	2	7	7
	2	2	7	7
	6	1	2	0,333
	2	3	20	30
	5	1	13	2,6
	2	2	15	15
	4	2	15	7,5
	5	2	10	4
	3	3	12	12
	2	3	15	22,5

2016

Number of initial potato varieties	Incubation period length (IPLi)	Sporulation intensity (SIi)	Lesion diameter (LDi)	Disease severity index (DSIi)
1	3	3	25	25
	3	3	20	20
	1	3	20	60
	3	2	10	6,667
	5	3	20	12
	4	3	20	15
	5	1	3	0,6
	5	2	20	8
	5	1	2	0,4
	4	2	12	6
	4	3	40	30
	4	2	7	3,5
	1	1	2	2
	1	2	22	44
	1	1	3	3
	4	3	10	7,5
371	7	1	2	0,286
		0	0	0
	6	1	3	0,5
		0	0	0
	7	1	4	0,571
	7	1	4	0,571
		0	0	0
		0	0	0
		0	0	0
		0	0	0
		0	0	0
		0	0	0
		0	0	0
	6	1	3	0,5
	8	1	1	0,125
		0	0	0
		0	0	0
		0	0	0
	7	1	2	0,286
		0	0	0
	7	1	3	0,429
	7	0	2	0
	6	1	2	0,333
	6	1	4	0,667
	7	1	2	0,286
	7	1	2	0,286
	7	0	2	0

2016 continued from page 9

372	7	1	3	0,429
	7	1	1	0,143
	6	1	3	0,5
	7	1	2	0,286
	7	1	1	0,143
	7	1	3	0,429
	7	1	3	0,429
	7	1	4	0,571
	7	0	0	0
	7	0	0	0
		0	0	0
		0	0	0
		0	0	0
	7	1	1	0,143
		0	0	0
		0	0	0
	7	0	2	0
	6	1	2	0,333
	5	1	3	0,6
	5	1	3	0,6
	7	0	1	0
	7	0	1	0
	5	1	10	2
376	2	2	14	14
	2	3	32	48
	6	1	4	0,667
		0	0	0
	4	1	22	5,5
	6	1	12	2
		0	0	0
	5	1	12	2,4
		0	0	0
	4	2	15	7,5
	4	2	10	5
	6	2	7	2,333
	7	1	1	0,143
	6	1	6	1
	5	1	7	1,4
		0	0	0
	6	1	5	0,833
		0	0	0
	5	3	6	3,6
	4	2	10	5
	6	2	7	2,333
	7	1	2	0,286

2016 continued from page 10

376	3	3	27	27
	4	1	1	0,25
	6	1	1	0,167
	7	1	1	0,143
377	7	1	2	0,286
	6	1	2	0,333
		0	0	0
	6	1	2	0,333
	6	1	2	0,333
	6	1	2	0,333
	6	1	5	0,833
	6	1	5	0,833
	5	1	6	1,2
	6	1	2	0,333
	6	1	2	0,333
		0	0	0
	6	1	2	0,333
	7	1	3	0,429
	5	1	3	0,6
	5	1	7	1,4
	3	1	20	6,667
	6	1	20	3,333
		0	0	0
	1	1	5	5
		0	0	0
		0	0	0
	7	0	1	0
		0	0	0
382	3	3	15	15
	3	3	20	20
	6	1	4	0,667
	3	2	20	13,333
	3	3	20	20
	4	2	13	6,5
	5	1	7	1,4
	4	2	10	5
	6	1	4	0,667
	5	2	30	12
	3	3	40	40
	3	3	35	35
	5	1	5	1
	3	1	30	10
	3	1	6	2
	6	1	3	0,5
	4	3	40	30

2016 continued from page 11

382	5	1	10	2
	2	3	20	30
	3	2	35	23,333
	7	1	3	0,429
	7	1	2	0,286
	3	1	7	2,333
	5	2	7	2,8
	4	1	5	1,25
	3	3	25	25
	7	1	4	0,571
387	6	1	5	0,833
	7	1	3	0,429
	5	3	10	6
	5	3	10	6
	2	3	32	48
	3	3	24	24
	6	1	5	0,833
	4	3	10	7,5
	5	1	10	2
	5	2	30	12
	4	2	30	15
	4	2	30	15
	8	0	2	0
	4	2	20	10
	5	2	17	6,8
	6	3	3	1,5
	5	1	20	4
	8	0	1	0
		0	0	0
	5	2	30	12
	6	2	10	3,333
	7	0	2	0
		0	0	0
	7	0	2	0
	5	2	20	8
	3	3	30	30
