# Supplementary material for: High Resistance of Potato to Early Blight Is Achieved by Expression of the Pro-SmAMP1 Gene for Hevein-Like Antimicrobial Peptides from Common Chickweed (Stellaria media)
Source: Plants (Basel). 2021 Jul 7;10(7):1395. doi: 10.3390/plants10071395 (PMC8309211; doi:10.3390/plants10071395)
Supplement: Supplementary file 1 [file plants-10-01395-s001.zip › Table S2.pdf]

Table S2. Correlation coefficients between either ProSmAMP1 expression or copy number and DSI, per year.

	correlation ProSmAMP1 expression - DSI		correlation ProSmAMP1 copy number - DSI	
year	Zhukovsky ranny	Udacha	Zhukovsky ranny	Udacha
2012	-0,56	-0,50	-0,81	-0,37
2014	-0,28	-0,67	-0,63	-0,64
2015/2016	-0,67	-0,10	-0,50	-0,55
mean	-0,50	-0,43	-0,65	-0,52
st dev.	0,20	0,29	0,16	0,14
