# Supplementary figures and images for: High Resistance of Potato to Early Blight Is Achieved by Expression of the Pro-SmAMP1 Gene for Hevein-Like Antimicrobial Peptides from Common Chickweed (Stellaria media)
Source: Plants (Basel). 2021 Jul 7;10(7):1395. doi: 10.3390/plants10071395 (PMC8309211; doi:10.3390/plants10071395)

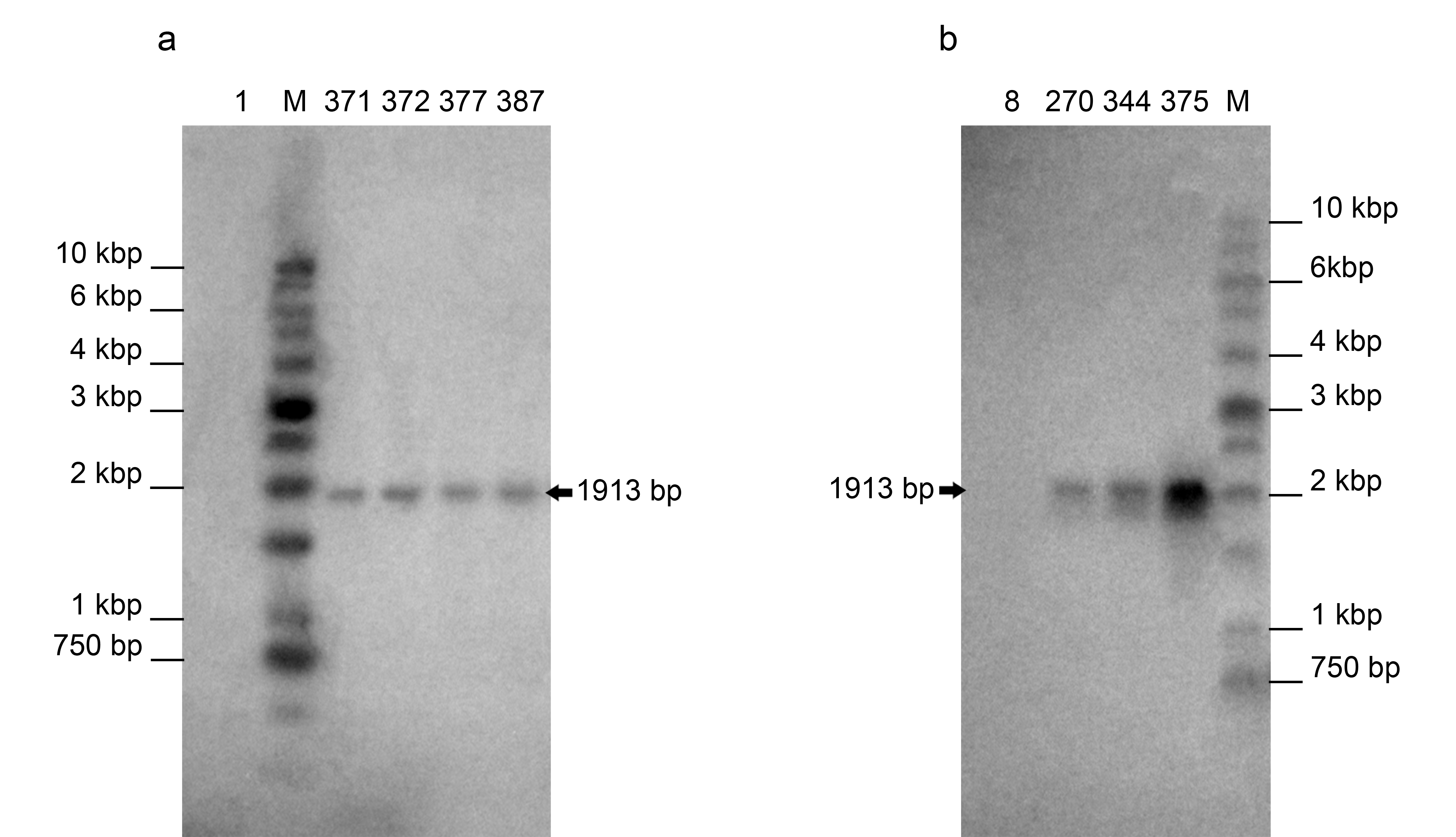

Supplement: Supplementary file 1 [file plants-10-01395-s001.zip › Figure S1.tif]
